# Occurrence, antibiotic susceptibility and genetic variation of *Streptococcus zooepidemicus* in Finnish weanling horses with and without respiratory infection

**DOI:** 10.1186/s13028-025-00839-0

**Published:** 2026-01-27

**Authors:** Reija Junkkari, Anna Mykkänen, Piia Sulku, Merja Rantala, Tarja Pohjanvirta, Marjut Eklund, Sinikka Pelkonen, Thomas Grönthal

**Affiliations:** 1 Department of Equine and Small Animal Medicine, University of Helsinki, Helsinki, FI-00014 Finland; 2 Horse Clinic Anivet, Raviraitti 45, Turku, FI-20380 Finland; 3 Research and Laboratory Department, Animal Health Diagnostic Unit, Finnish Food Authority, Neulaniementie 4, Kuopio, FI-70201 Finland

**Keywords:** Antimicrobial susceptibility, Genetic variation, Respiratory infection, S. zooepidemicus, Weanling horse.

## Abstract

**Background:**

*Streptococcus zooepidemicus* subsp. *zooepidemicus* (*S. zooepidemicus*), an opportunistic pathogen often found in the stable environment and upper respiratory tract of young horses, can cause severe pneumonias in Equidae. In this study we investigated the occurrence, genetic variation and antimicrobial susceptibility of *S. zooepidemicus* isolates from 63 weanling horses kept in loose housing or conventional stables. The bacterial isolates were typed by using pulsed-field gel electrophoresis (PFGE) and multi-locus sequence typing (MLST). The possible associating factors (stable type, age, breed and clinical signs) for positive *S. zooepidemicus* finding were analysed using logistic regression analysis. In addition, we describe antimicrobial susceptibility of 535 equine *S*. *zooepidemicus* isolates in Finland derived from clinical samples sent to the Clinical Microbiology Laboratory of the Faculty of Veterinary Medicine, University of Helsinki from 2011 to 2024.

**Results:**

Out of the 63 weanling foalssampled, *S. zooepidemicus* was isolated in 26 (41%). There was a positive correlation between signs of respiratory infection and positive *S. zooepidemicus* finding (OR = 5.15, 95% CI: 1.169–22.694). Age was also a significant factor (OR = 0.972, 95% CI: 0.952–0.992), as younger foals were more likely to be *S. zooepidemicus* positive. Bacterial isolates were distributed into eleven PFGE clusters. The largest cluster contained six isolates, followed by clusters with five and three isolates, respectively. The cluster with three isolates appeared to have a new *pro*S allele in MLST analysis. In addition, two more new sequence types were observed. Sequence types observed in our study differed from those previously identified in two earlier equine studies conducted in Sweden and Iceland. All isolates from weanling foals in our study were susceptible to penicillin and trimethoprim-sulfamethoxazole. There was no resistance to penicillin in the patient samples (*n* = 535) from 2011 to 2024. Of the 522 isolates tested, 28 (5.4%) were classified as either resistant or intermediate to trimethoprim-sulfamethoxazole. Only 24% of the isolates tested (*n* = 403) were susceptible to tetracycline.

**Conclusions:**

Younger weanlings are more likely to have a positive *S. zooepidemicus* finding and their clinical signs, including high temperature, are associated with this common opportunistic pathogen. A high level of genetic variation of *S. zooepidemicus* is evident in weanling horses. Based on our findings, there is no evidence to suggest that *S. zooepidemicus* exhibits reduced susceptibility to penicillin. Additionally, the susceptibility to trimethoprim-sulfamethoxazole in the horses under study remains high.

## Background

*Streptococcus equi* subsp. *zooepidemicus* (*S. zooepidemicus*) is a β-hemolytic streptococcus that is often found as part of the normal microbiome in the upper respiratory tract of horses [1] and also in the stall environment [2]. *S. zooepidemicus* can also be a primary pathogen and cause pneumonia in horses [3–7]. Some findings suggest that there is variability in pathogenicity of *S. zooepidemicus* as different strains have been isolated from clinically ill opposed to clinically healthy horses during a respiratory outbreak [4, 5].

In addition to species in the family Equidae, *S. zooepidemicus* can infect multiple other species. Outbreaks of respiratory infections have been described in pigs, dogs, cats, and guinea pigs, especially in dense populations [8–12]. *S. zooepidemicus* can cause fatal outbreaks in pigs and there is a marked difference in virulence between the strains [13]. In humans, *S. zooepidemicus* can cause both respiratory tract and severe systemic infections. Infection can be acquired through consumption of unpasteurized dairy products [14] or having close contact with infected dogs [15], guinea pigs [16] or horses [17–19]. Infections have been described in men aged over 50 years with pre-existing conditions such as cardiac disease or diabetes and who work with horses [17].

In respiratory outbreaks, identification of bacterial species and characterization of the strain responsible for the outbreak is often important for identifying the source of infection. Multi-locus sequence typing (MLST) is a molecular technique that is based on sequencing highly conserved housekeeping genes and allows the comparison of bacterial isolates. The sequence data is comparable between laboratories and therefore this technique enables global epidemiological surveys [20]. For the *S. zooepidemicus* group, an MLST technique was developed based on the sequencing of seven housekeeping genes (*arc*C, *nrd*E, *pro*S, *spi*, *tdk*, *tpi* and *yqi*L) [21]. Based on these allelic sequences the strains are classified into sequence types (STs). Sequence type 24 was the causative agent in a Swedish outbreak in 2009 [4] and sequence type 209 in Iceland in 2010 [5]. Both these outbreaks affected Icelandic horses. ST 209 has also been recovered from human patients [17, 22].

In Finland, weanling horses, especially trotters, are gathered in groups from different farms in the late autumn and kept in loose housing systems until the following summer. Based on the results of our observational field study [23] it seems that the younger weanlings born late in the season are more likely to have signs of respiratory disease when gathered together after weaning. In order to design an appropriate treatment strategy, it is essential to accurately identify the causative pathogen. The causative agent in equine pneumonia varies by age, where *S. zooepidemicus* is a common cause of pneumonia in weanling horses [3, 24]. *S. zooepidemicus* is still considered to be generally susceptible to penicillin [7], however, antimicrobial resistance situation should be closely monitored [25, 26].

The first aim of this study was to investigate the occurrence, genetic variation and antimicrobial susceptibility of the *S. zooepidemicus* strains collected during our earlier study [23]. The second aim was to investigate if there is an association between a genetic profile of *S. zooepidemicus* and clinical signs and to identify potential associating factors for the occurrence of *S. zooepidemicus*. Our third aim was to obtain a broader overview of the antimicrobial resistance profile of equine *S. zooepidemicus* isolates in Finland by analysing the antimicrobial susceptibility of isolates found in equine samples sent to the Laboratory of Clinical Microbiology at the Faculty of Veterinary Medicine, University of Helsinki (YESLAB) from 2011 to 2024.

## Methods

### Study population and clinical examination

The original study population included 70 weanling foals, but seven were excluded from the study because of missing data on the clinical signs. The final study population included 63 weanling foals from six loose housing (LH) and five stable (SF) farms located in Eastern Finland. The Finnish Equine Information Centre (Hevostietokeskus) – a national expert and research organization – conducted a regional survey to identify potential participants. Farms were included in the study based on the owners’ willingness to participate, using a convenience sampling method. Weanlings in the loose housing farms were kept in a group and were able to move freely between paddock and shelter. Adult horses were not located in the same shelters or paddocks with the weanlings in the loose housing farms. In the stable farms, weanlings were kept in individual stalls inside a barn and the stall doors opened to the barn aisle. Adult horses were housed in the same barn with the weanlings. The weanlings spent the nights in the barns and were turned out into a paddock in a group during the daytime.

The study was conducted in two phases: in 2013–2014 and 2014–2015. During each phase, the farms were visited between November and January. The farms were visited three times during our earlier study [23]. Sample results and information collected at the first visit were used for statistical analysis. The horses were clinically examined by a veterinarian at the farm visit prior to sampling. Special attention was paid to the signs of respiratory disease, which were temperature ≥ 38.3 °C and one or more of the following signs: cough, nasal/ocular discharge or increased respiratory sounds [27, 28].

## Sample collection and bacterial culture

Nasal swabs (M40 Transystem Amies Agar Gel, without charcoal, Copan Diagnostics, CA, USA) for bacterial culture were collected from the nostrils. The samples were stored at + 4 °C and cultured within 48 h after the sampling. For isolate identification, Gram stain, APIStrep (bioMérieux, France) test and agglutination with streptococcal group sera (streptococcal Grouping Kit; Oxoid, Basingstoke, UK) were carried out. The initial bacterial culture and identification was done by the Finnish Food Authority, Kuopio, Finland [17], after which the strains were stored at − 80 °C until further analysis. For historical data, identification of *S. zooepidemicus* was based on Lancefield grouping and a short sugar series [29] prior to 2017. After this, identification was done by Matrix-Assisted Laser Desorption Ionization Time-of-Flight (MALDI-ToF) analysis using the Bruker Microflex LT device and the direct smear method.

### Molecular typing of the bacterial isolates

#### Pulsed-field gel electrophoresis (PFGE)

To obtain an overview of the heterogeneity of circulating *S. zooepidemicus* strains, the isolates collected in the course of our earlier study [23] were typed with pulsed-field gel electrophoresis (PFGE). Altogether 28 bacterial isolates were further typed. These included 26 isolates from the loose housing farms (LH5: eleven isolates, LH3: eight isolates, LH4: three isolates, LH1 and LH2: two isolates, LH1: one isolate) and two isolates from two different stable farms (SF6 and SF7: one from each farm). *Sma*I (New England Biolabs, MA, USA) restriction enzyme patterns of the isolates and control strains were investigated according to a published protocol for *Streptococcus pneumoniae* [30], with minor modifications: bacterial suspensions of 8.0–9.0 McFarland density were embedded in SeaKem Gold (Lonza, ME, USA) agarose. PFGE was performed on a Chef DR III system (Bio-Rad, CA, USA). The total run time was 16 h in + 14 °C; the first-block switch time was 2.2 s and the final switch time 35 s. The voltage for the run was 6 V/cm with an included angle of 120°. SYBR Safe (Invitrogen, CA, USA) was used to stain the DNA fragments that were visualized with the AlphaImager (Alpha Innotech, CA, USA) system. GelCompar II software (v. 6.6 Applied Maths, Belgium) was used to examine PFGE fingerprints. The PFGE cluster analysis was made by UPGMA, using the Dice similarity coefficient, and optimization and position tolerance were both set at 1.5%. Clonal clusters were determined using 85% similarity cut-off [31]. Based on the PFGE analysis, representative isolates from each cluster were selected for MLST analysis.

### Multilocus sequence typing (MLST)

Bacterial isolates were selected for MLST analysis based on PFGE results. Isolation of the DNA from bacterial cells was performed according to the manufacturer’s instructions using InstaGene Matrix (Bio-Rad) and the supernatant was used as a PCR template. The MLST typing was performed according to the international MLST scheme for *S. zooepidemicus* [32] using Phusion Flash (Thermo Fisher Scientific, MA, USA) enzyme. The PCR products were purified with ExoI and FastAP protocol according to the manufacturer’s instructions (Thermo Fisher Scientific). Sanger sequencing was performed at a commercial laboratory (Macrogen, Netherlands) with an ABI 3730 XL automated sequencer. The sequences were analysed with the CLC Main Workbench software (version 23, Qiagen, Denmark).

Antimicrobial susceptibility.

Antimicrobial susceptibility of the bacterial isolates collected at the first visit [23] was tested according to Clinical and Laboratory Standards Institute (CLSI) standard [33, 34] using Kirby-Bauer disc diffusion for penicillin and ETEST MIC testing according to manufacturer’s (bioMérieux) instructions for trimethoprim-sulfamethoxazole. The standard in effect at the time of each analysis was applied.

Susceptibility results for bacterial isolates from equine specimens (*n* = 535) were retrospectively analysed by searching through the YESLAB laboratory information system (Provet Net, Nordhealth, Finland) database for all equine isolates of *S. zooepidemicus* from 2011 to 2024. Disk diffusion susceptibility testing was conducted following the CLSI guidelines [33, 34] valid at the time. The different guideline versions used during the study did not differ substantially.

### Data analysis

The percentage of foals (*n* = 63) from which *S. zooepidemicus* was isolated was calculated. Possible associating factors (gender, stable type, breed, clinical signs of respiratory infection, and age) were first assessed with univariable logistic regression, each risk factor separately as the sole explanatory factor. The factors in the univariate logistic regression found to be meaningful (P*-*value < 0.1) were inserted into a multivariate logistic regression model. Odds ratios (OR) with 95% confidence intervals (CI) were calculated to quantify the results. P-values < 0.05 were considered statistically significant. Statistical analyses were performed using IBM SPSS Statistics for Windows, Version 29.0 (IBM, NY, USA).

## Results

**Fig. 1:**
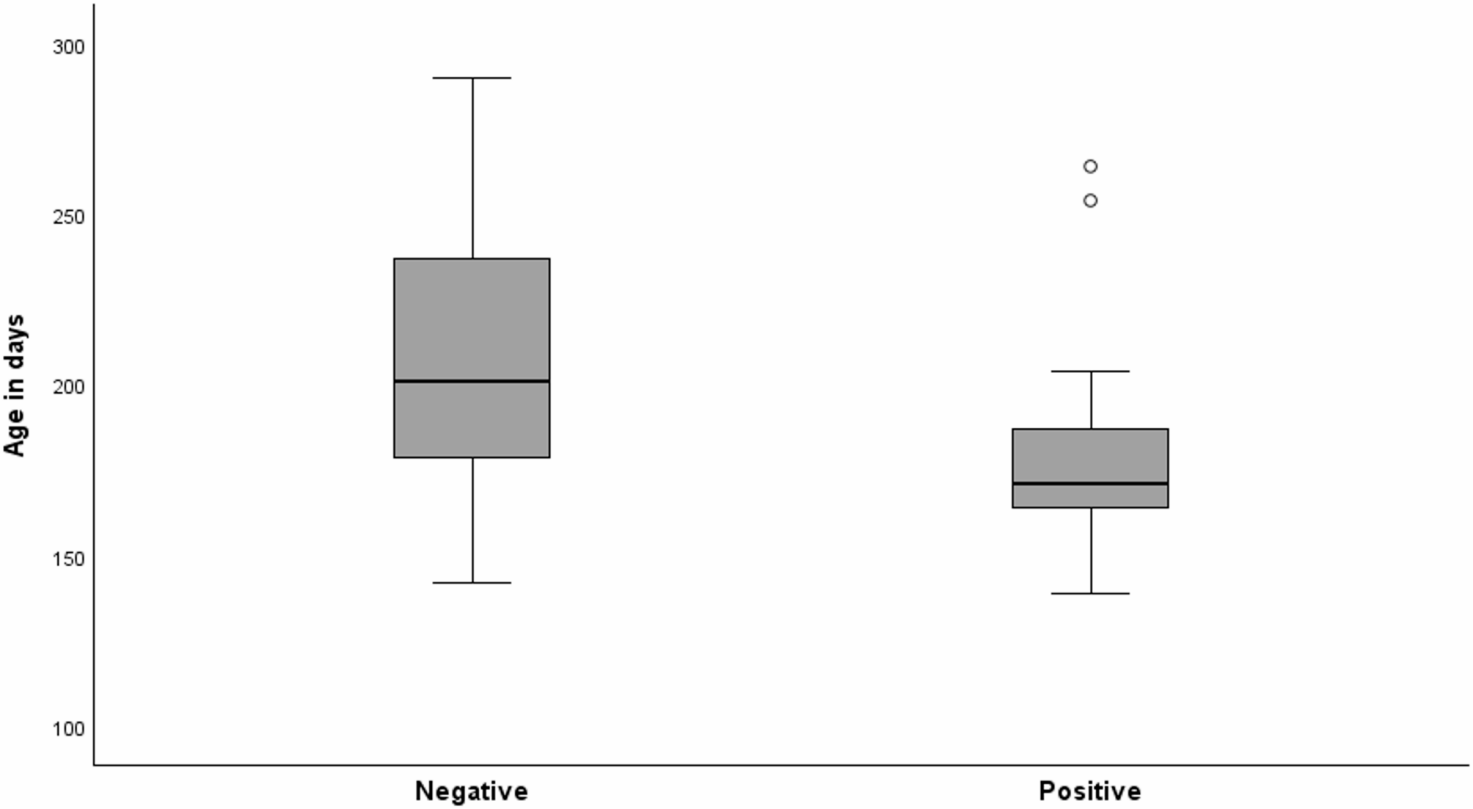
The age distribution of horses (*n* = 63) with positive or negative bacterial culture. The nasal swap samples were taken in two phases in 2013–2014 and 2014–2015 at the farm visits in Eastern Finland. The number of horses with positive *Streptococcus equi* subsp. *zooepidemicus* finding was 26 and the number of horses with negative culture result was 37

Out of the 63 horses sampled at the first visit, 12 foals had signs of respiratory disease and *S. zooepidemicus* was found in 26 (41%). The weanlings were 139–290 days old (mean, 197; median 187) at the time of the examination. Weanlings with a negative culture had a mean age of 209 days, compared to 178 days among those with a positive test result (Fig. 1).

**Fig. 2:**
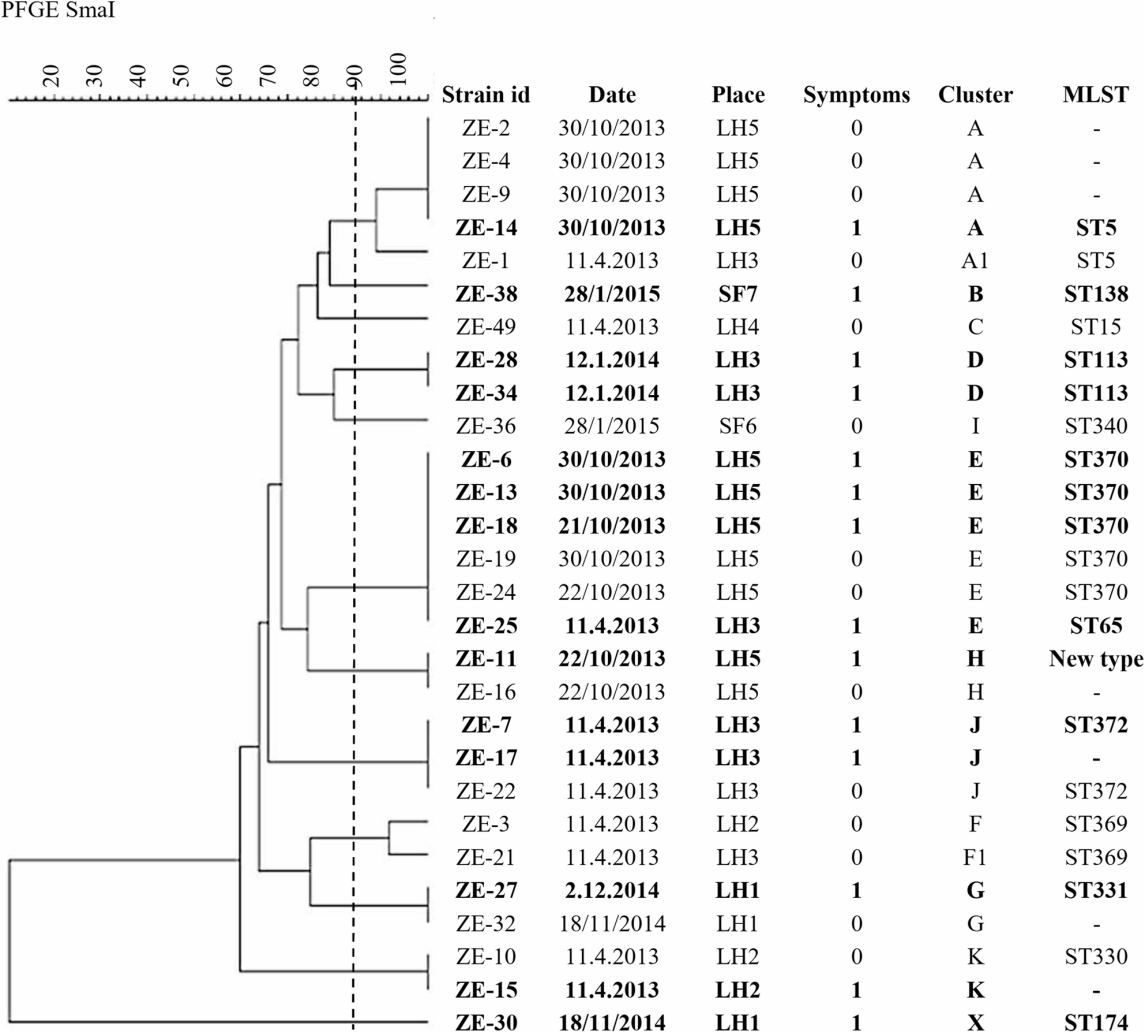
Dendrogram of 28 *Streptococcus equi* subsp. *zooepidemicus* isolates from weanling horses in Eastern Finland. Date = sampling date, LH = loose housing farm, SF = stable farm. Strains with bold font are isolated from horses with signs of respiratory disease. The dashed vertical line indicates the 85% similarity cut-off

PFGE was performed for 28 isolates, two of which were obtained from foals that were excluded from the statistical analysis because of missing data (ZE-14: temperature) and euthanasia (ZE-21: cachexia, anaemia, heart murmur, dehydration). MLST was performed for 21 representative isolates of each PFGE-cluster. *S. zooepidemicus* isolates were distributed into 11 PFGE clusters (Fig. 2). One isolate (ZE-30) was not typeable using *sma*I restriction enzyme. The largest cluster (E) had six isolates followed by cluster A with five isolates. Cluster J had three isolates. Of the six cluster E isolates, five were from the same farm (LH5) and of a new sequence type (ST370). The remaining isolate, ST65, was from another farm (LH3) and was a triple locus variant of ST370, and is the closest ST to it in the MLST database. The isolates of cluster A were of ST5 and were from two different farms (LH5 and LH3). Isolates from cluster J were all from the same farm (LH3) and had a novel *pro*S allele (ST372). In addition, one more new type with non-amplifiable *pro*S gene (*arcC* 3; *nrd*E 3; *pro*S negative; *spi* 66; *tdk* 10; *tpi* 18, *yqiL* 30) was observed in cluster H. This is closest to, and a single locus variant of, ST373, a sequence type described from an equine nasal swab in Finland. Of the remaining seven STs (ST15, ST113, ST138, ST174, ST330,ST331, and ST340) all except ST174 and ST331 (both at LH1) occurred in different farms (Fig. 2). No epidemiological link between the different premises was identified in this study.

In the multivariable model, age (*P* = 0.007) and the presence of clinical signs of respiratory infection at the time of sampling (*P* = 0.030) were predictors of a positive *S. zooepidemicus* finding. When temperature is left out from the criteria, the number of sick animals increases from 12 to 24 out of 63. Subsequently the chi-square test shows no association between positive bacterial finding and clinical disease (without fever) *P* = 0.206. Age was negatively associated with the outcome, while animals with clinical signs had significantly higher odds of testing positive (Table 1).

**Table 1:** Univariable and multivariable logistic regression analysis for the probability of positive *S. zooepidemicus* sample

	Univariable logistic regression		Multivariable logistic regression	
Variables	OR (95% CI)	*P*	OR (95% CI)	*P*
Breed	2.275 (0.695; 7.449)	0.174		
Gender	1.516 (0.547; 4.204)	0.424		
Stable type	3.875 (0.759; 19.613)	0.104		
Clinical signs	6 (1.435; 25.084)	0.014	5.150 (1.169; 22.694)	0.030
Age	0.972 (0.953; 0.990)	0.003	0.972 (0.952; 0.992)	0.007

The factors with P-value < 0.1 in the univariable analysis were included in the multivariable model

**Fig. 3:**
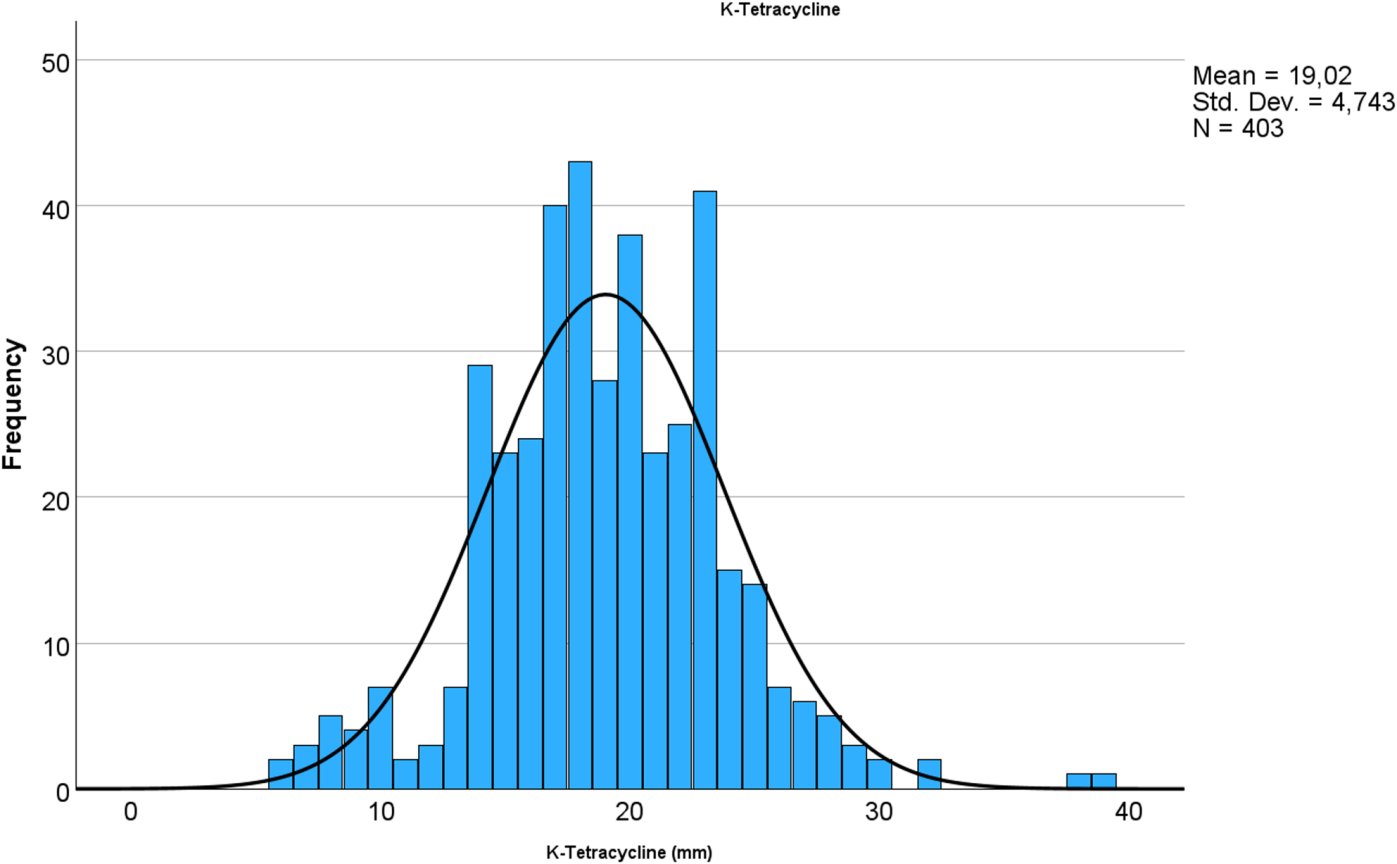
Distribution of tetracycline disk-diffusion susceptibility results in 403 equine *S. zooepidemicus* isolates. The isolates were analysed in YESLAB in 2011–2024. Vertical bars indicate clinical breakpoints for resistant and susceptible population

**Fig. 4:**
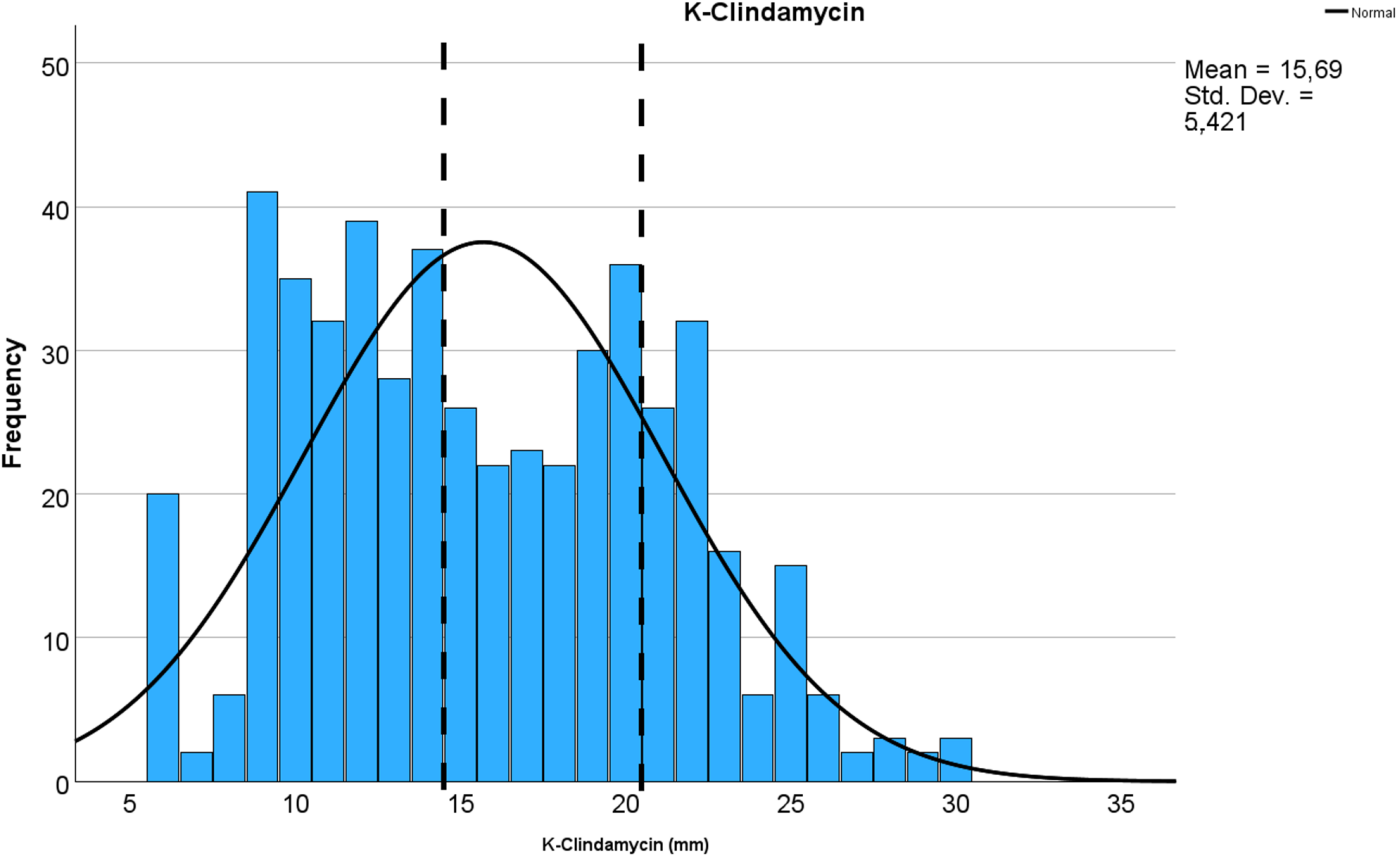
Distribution of clindamycin disk-diffusion susceptibility results in 510 equine *S. zooepidemicus* isolates. The isolates were analysed in YESLAB in 2011–2024. Vertical bars indicate clinical breakpoints for resistance and susceptible population

The bacterial isolates collected in the stable visits (nasal swab, *n* = 28) in 2013–2014 and 2013–2015 were susceptible to penicillin and trimethoprim-sulfamethoxazole. For trimethoprim-sulfamethoxazole, E-test MIC_50_ and MIC_90_ were 0.047 and 0.064 mg/L, respectively. All equine *S. zooepidemicus* isolates (*n* = 522; bronchoalveolar lavage (BAL) or tracheal aspirate or wash: 213; other respiratory sample: 57; deep pus specimens and fluid aspirates: 165; superficial pus samples: 56; surgical site infections: 20; sample type not specified: 6) analysed in 2011–2024 were susceptible to penicillin. Susceptibility to tetracycline and clindamycin appeared very low (Table 2). However, clindamycin or tetracycline histograms were not typical for acquired resistance (Figs. 3 and 4).

**Table 2:** The antimicrobial susceptibility of equine *S. zooepidemicus* isolates (*n* = 535) analysed in YESLAB in 2011–2024. S = susceptible, I = intermediate, R = resistant

Antimicrobial	*n*	S	I	*R*
Penicillin G	522	100% (522)	0	0
Trimethoprim- Sulfamethoxazole	522	94.6% (494)	3.3% (17)	2.1% (11)
Erythromycin	510	98.8% (504)	1.2% (6)	0
Clindamycin	510	22.4% (114)	31,4% (160)	46.3% (236)
Tetracycline	403	24.0% (97)	28.3% (114)	47.6% (192)

## Discussion

This study investigated the occurrence, risk factors, genetic diversity, and antimicrobial susceptibility of *S. zooepidemicus* in Finnish weanling horses housed in either loose housing systems or conventional stables. Unlike previous studies that focused on outbreaks, our approach involved sampling of all foals on selected farms, regardless of clinical signs. This method provided a snapshot of the endemic presence and genetic variability of *S. zooepidemicus* in the selected loose housing and stable farms located in Eastern Finland.

The presence of clinical respiratory disease signs was associated with a positive *S. zooepidemicus* finding and younger foals were more likely to show clinical signs of respiratory infection. This supports previous findings that age-related immunity plays a role in susceptibility to *S. zooepidemicus* infection [3, 23]. In this study, clinical disease was defined as either high fever alone or fever in combination with other clinical signs. When fever was excluded and only other signs were considered, the proportion of foals classified as sick doubled. Using this definition, however, *S. zooepidemicus* findings were no longer associated with the presence of clinical signs. In some foals, high fever was the only manifestation at the time of sampling. Based on these findings, frequent temperature monitoring can still be recommended for the surveillance of respiratory health in young horses. The type of housing (loose housing vs. conventional stable), gender or breed, were not associated with the presence of *S. zooepidemicus*.

PFGE analysis of 28 isolates revealed significant genetic heterogeneity, with isolates divided into 11 clusters. Although some farms showed local clonal spread (e.g., cluster E in farm LH5), most STs were genetically distinct, including the isolates from the same farm. These findings are similar to a study from central Italy where nasal swabs from 478 adult horses from 99 different farms were analysed. Fifty-six were culture positive, 31 different STs and 14 new STs were found [1]. As in our study, they also noticed that the younger horses were more likely to test positive: the median age of positive animals in their study was 6.5 years and, and 10 years in negative animals. We found no association between the clinical signs of respiratory disease and bacterial strain. Our results indicate that pathogenicity may not be restricted to specific isolates but could instead be influenced by host-related factors. Interestingly, Cito et al. [1] noticed that donkeys were more likely to test positive compared to horses. In two earlier studies, ST24 [4] and ST209 [5] were identified in respiratory outbreaks, but neither were found in this study. Two novel STs were identified, however. As *S. zooepidemicus* can survive up to three days on concrete or wood surfaces in farm-like conditions [35], it is possible that the foals may acquire the infection from the environment upon arrival, although transmission from another weanling is more likely. When salt water obtained from an equine hydrotherapy unit was studied, minimal to no reduction in bacterial concentrations of *S. zooepidemicus* at 2 °C over the duration of the 96-hour study was detected [36] and in another study drinking from contaminated water sources was recognised as a risk factor for *S. zooepidemicus infection* [37]. It could therefore be possible that the infection might also spread through water bowls that are shared in the loose housing systems and also in stable farms when horses are turned out. Therefore, water bowls should be emptied and cleaned frequently.

This study did not reveal any epidemiological link between the different farms. However, as no signs of an outbreak were observed during our visits to the farms, no epidemiological investigation was conducted, nor were questions asked regarding possible human movement between the farms.

Our study provides insights into the occurrence and antimicrobial susceptibility patterns of *S. zooepidemicus* in young horses under field conditions in Finland. Antimicrobial susceptibility results were consistent with previous findings from Nordic countries [38]. All isolates tested between 2011 and 2024 (*n* = 522) were susceptible to penicillin G, supporting its continued use as the first-line treatment for *S. zooepidemicus* infections when antimicrobials are needed. High susceptibility was also observed for trimethoprim-sulfamethoxazole and erythromycin. Susceptibility results of *S. zooepidemicus* isolates revealed that a large proportion appeared non-susceptible to clindamycin and tetracycline. Considering that all but six *S. zooepidemicus* isolates were susceptible to erythromycin, and considering prevailing acquired macrolide resistance mechanisms in beta haemolytic streptococci, mainly *erm*- or *mef*-genes and less frequently ribosomal mutations, clindamycin result was not expected. We did not find clindamycin disk diffusion histograms for wild type *S. zooepidemicus* isolates from public sources such as EUCAST, but clindamycin MIC distribution range for wild type isolates (with only 50 observations, though) is 0.006-1 mg/L [39]. However, unpublished data from Finnish Food Authority reference laboratory revealed similar wide distribution of zone diameters (from 11 to 26 mm) as in our study with clindamycin disk diffusion test for this species, but the number of tested isolates was less than 30. Nevertheless, clindamycin disk diffusion histogram of our study is not typical for acquired macrolide-lincosamide resistance indicating that clindamycin disk diffusion test may not be suitable for this bacterial species. Further research is warranted, e.g. by testing clindamycin parallel with MIC and disk diffusion methods for both *S. equi* subspecies.

For tetracycline, while the zone diameters were normally distributed, the human derived breakpoints were in the middle of the distribution, suggesting that the reduced susceptibility observed does not reflect acquired resistance, but that *S. zooepidemicus* is intrinsically less susceptible to tetracycline. This is supported by the tetracycline MIC distribution of *S. zooepidemicus* in the EUCAST database [39]. However, human-derived clinical breakpoints may not be suitable for animals, highlighting the need for veterinary-specific breakpoints. EUCAST is currently working towards the development of such animal-specific interpretive criteria (VetCAST).

The reason we tested erythromycin and clindamycin is for resistance surveillance purposes only. In horses, erythromycin or clindamycin are not among treatment options for streptococcal infections. However, since *S. zooepidemicus* is a zoonotic organism, we consider that resistance surveillance for macrolides and lincosamides is warranted. Further studies are needed to clarify these findings, as confidence in susceptibility testing methods is essential for guiding appropriate antimicrobial use. On the other hand, susceptibility testing should be done only for acquired resistance, not intrinsic resistance. Our findings highlight the need for susceptibility testing prior to treatment, when considering alternatives to beta-lactam antibiotics.

This study had several limitations. Sampling was done in 2013–2015 as part of ourprevious research, so the data may not accurately reflect the current situation. Also, the fact that samples were collected from the nostrils, although some of the weanlings had increased respiratory sounds, leaves uncertainty about the role of *S. zooepidemicus* in lower airway disease in these individuals. Additionally, the timing of sampling was dictated by logistical constraints, which may have resulted in underrepresentation of acute infections, if the infection had already been healed at the time of the visit. On the other hand, when fever was excluded from the clinical signs, the presence of *S. zooepidemicus* was no longer associated with clinical disease. This suggests that *S. zooepidemicus*, particularly when isolated from the upper respiratory tract, may be associated with milder clinical manifestations. Further studies using lower airway sampling (e.g. tracheal wash) with broader geographic representation are warranted to better understand the pathogenic role and transmission dynamics of *S. zooepidemicus* in weanling foals.

## Conclusions

Our findings highlight the widespread presence and genetic diversity of *S. zooepidemicus* across farms in weanling horses, and no evidence of dominant epidemic clones. The association between clinical respiratory signs and positive cultures, combined with age-related susceptibility, underscores the importance of host factors in disease development. Younger weanlings have a higher risk of a positive *S. zooepidemicus* result. *S. zooepidemicus* isolates from weanling foals, as well as those found in equine samples in 2011 to 2024, were highly susceptible to penicillin and trimethoprim-sulfamethoxazole.

Penicillin remains highly effective, reaffirming its role as the first-choice antibiotic in equine respiratory disease.

## Data Availability

The datasets used and/or analysed during the current study are available from the corresponding author upon reasonable request.

## Declarations

### Prior publication

Data have not been published previously.
